# Predictors of poor outcome in cervical spondylotic myelopathy patients underwent anterior hybrid approach: focusing on change of local kyphosis

**DOI:** 10.1186/s13018-020-01905-1

**Published:** 2020-08-31

**Authors:** Xing Jian Cheng, Lin Jin, Xin Wang, Wen Zhang, Yong Shen

**Affiliations:** 1 Department of Orthopedic Surgery, The Third Hospital of Hebei Medical University, No. 139 Ziqiang Road, Shijiazhuang, 050051 China; 2 Department of Trauma Emergency Center, The Third Hospital of Hebei Medical University, No. 139 Ziqiang Road, Shijiazhuang, 050051 China; 3 Department of Foot and Ankle Surgery, The Third Hospital of Hebei Medical University, No. 139 Ziqiang Road, Shijiazhuang, 050051 China

**Keywords:** Cervical spondylotic myelopathy, Local kyphosis, Hybrid approach, T1 slope angle, Postoperative outcome

## Abstract

**Objective:**

This study was a retrospective multivariable analysis for risk factors of poor outcome in patients who underwent anterior hybrid approach, and discussed the causes of worsening of postoperative local alignment.

**Methods:**

A total of 86 patients with progressive spinal cord compression and local kyphosis underwent an anterior hybrid approach (ACDF+ACCF), between June 2011 and June 2017. We evaluated clinical outcome by the Japanese Orthopaedic Association (JOA) score and recovery rate. Patients were divided into two groups according to the worsening and improving of postoperative local alignment. Multivariate logistic regression analysis was applied to the evaluation of risk factors. Mann-Whitney *U* test, independent *t* test, and chi-squared test were performed for the comparison of local kyphosis between postoperative and last follow-up.

**Results:**

There were twenty patients who had a recovery rate of less than 50%. Advance age, longer duration of symptoms, bigger T1 slope angle, and lower change of local kyphosis angle were significantly associated with a poor clinical outcome by multivariate logistic regression analysis. The cause of worsening of postoperative local alignment had T1 slope, C2–7 sagittal vertical axis (SVA), adjacent segment degeneration (ASD), and implant subsidence.

**Conclusions:**

The change of local kyphosis was a predictor of clinical outcome after the hybrid approach. Furthermore, postoperative ASD, implant subsidence, T1 slope, and C2–7 Cobb were associated with recurrence of postoperative cervical kyphosis.

## Introduction

Cervical spondylotic myelopathy (CSM) as a result of cervical stepwise degeneration usually has a deterioration of neurological function. In some patients with CSM, the quite formation of cervical kyphosis (CK) owed to biomechanical force change and tonic muscular activity [1]. Progressive cervical kyphosis could lead to cervical pain, lower quality of life, and decrease of cord volume in the spinal canal [2–5]. Although conservative treatment could improve this disease to a certain extent, surgery remained the key to decompression of the spinal cord and correction of kyphosis [6–8]. The approaches of treatment of multilevel CSM (m-CSM) with CK included anterior cervical discectomy and fusion (ACDF), anterior cervical corpectomy and fusion (ACCF), and posterior laminectomy and laminoplasty. Both anterior and posterior approaches were able to improve the clinical recovery of neurological function, but the selection of approaches remained controversial [9, 10].

With the improvement of medical apparatus and surgeon’s skill level, the difficulty of anterior approaches and postoperative complications is gradually decreasing. Because of the efficacy of anterior approaches with directly decompressed spinal cord and great correction of CK [11], a hybrid approach (ACDF+ACCF) was applied to the treatment of patients with m-CSM and CK. Nevertheless, few studies have focused on the analysis of risk factors of surgical outcomes in patients after correction of kyphotic alignment by the hybrid approach. The aim of our study was to assess the relationship between improvement of kyphotic deformity and recovery of neurological function, particularly the changes of local kyphotic angle. Furthermore, the causes of worsening of postoperative local alignment were investigated deeply using statistical analyses. Our findings could offer advices to surgeons, thereby achieving optimal surgical outcome.

## Material and methods

### Patient population

Between June 2011 and June 2017, eighty-six patients with m-CSM and cervical kyphosis underwent a hybrid approach which includes one level ACDF and one level ACCF. All procedures were approved by the Ethics Committee of The Third Hospital of Hebei Medical University, and the written informed consent was acquired from each patient. The STROBE statement was strictly observed over the course of this study.

The criteria were defined as follows: (1) patients who were diagnosed with m-CSM and cervical kyphotic deformity; (2) ossification of the posterior vertebral body, disc herniation, and kyphotic alignment contributed to the spinal cord compression; (3) patients had progressive clinical symptoms with or without increased signal intensity of the spinal cord on T2-weighted magnetic resonance imaging (MRI); and (4) each case underwent the hybrid approach for decompression of the spinal cord and correction of CK. Some exclusion criteria were defined as follows: (1) patients had cervical surgery before, (2) patients with spinal tumors or congenital cervical deformity or peripheral nerve disease, and (3) patients with incomplete imaging data.

### Neurological assessment

In this study, each individual patient had a follow-up over 12 months, in which neurological function was evaluated by using the Japanese Orthopaedic Association (JOA) score before surgery and last follow-up. The time period of optimum recovery usually occurred within 12 months after surgery [12]. The surgical outcome was based on the following formula: the recovery rate = (postoperative JOA score − preoperative JOA score)/(17 − preoperative JOA score) × 100%. A score ≥ 75% was defined as excellent, ≥ 50% but < 75% as good, ≥ 25% but < 50% as fair, and < 25% as poor. Therefore, we divided recovery rates < 50% and ≥ 50% into the poor group and good group, respectively.

### Radiologic assessment

**Fig. 1:**
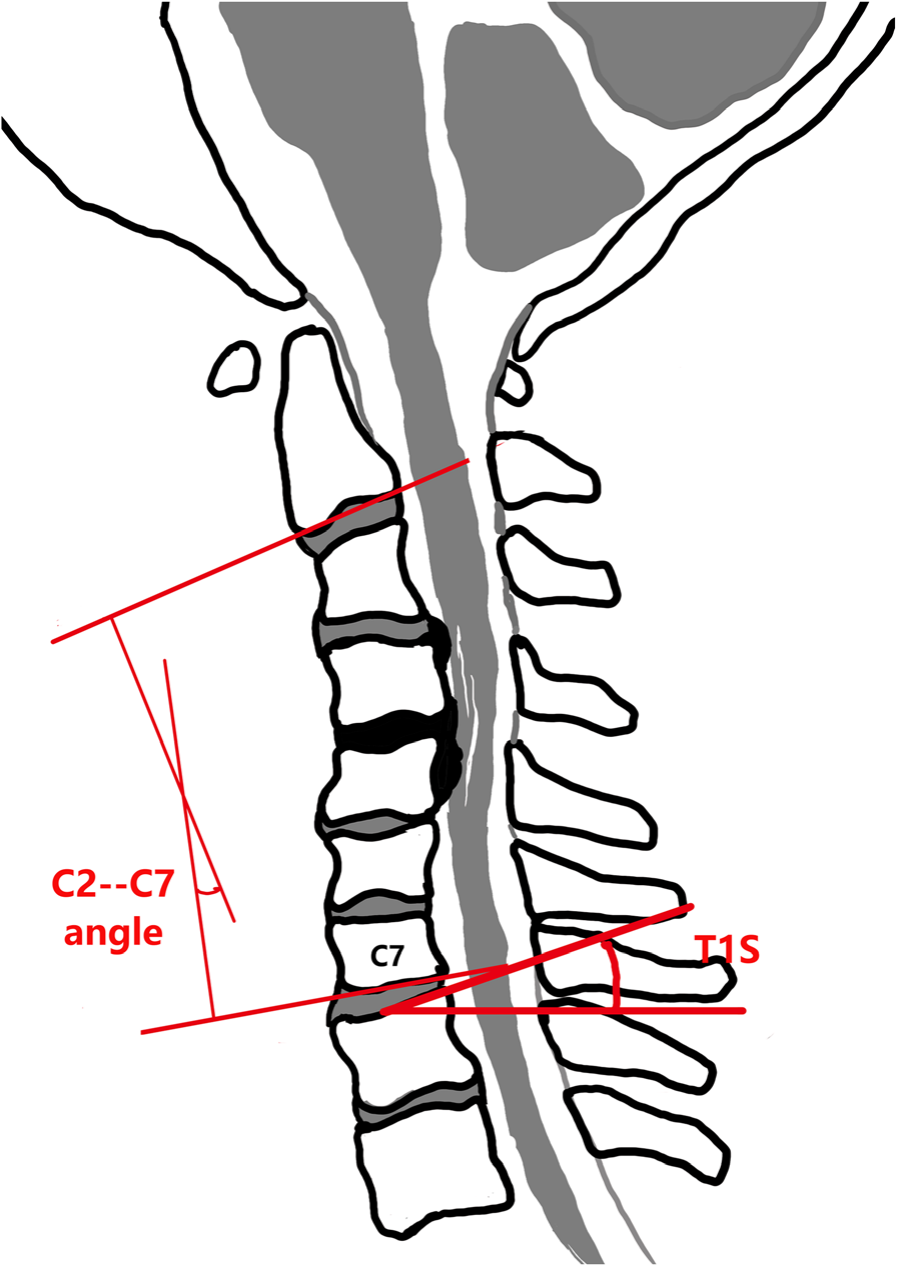
A schematic drawing: patients with cervical spondylotic myelopathy and cervical kyphosis. Radiographic measurements: T1 slope and C2–7 Cobb angle

**Fig. 2:**
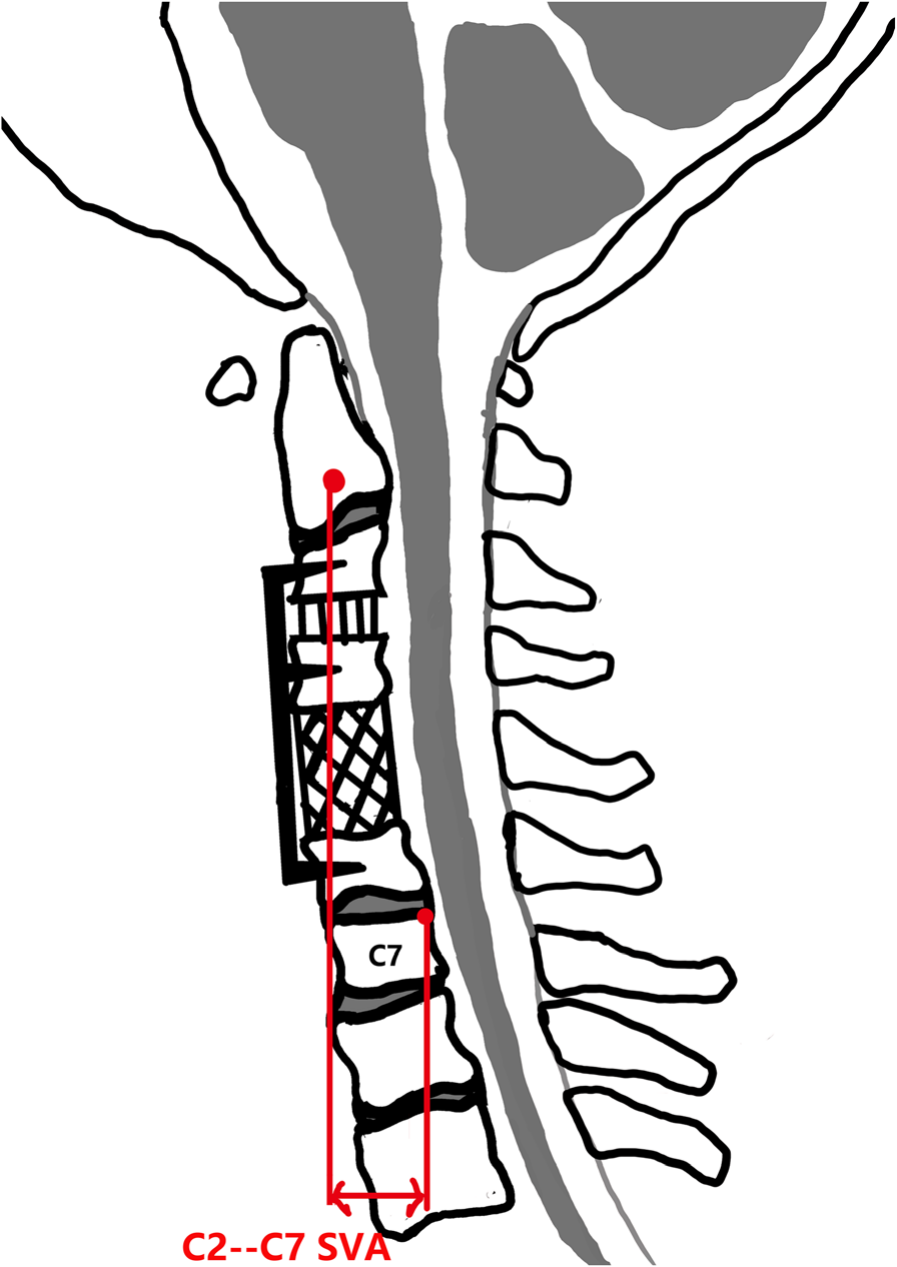
A schematic drawing: patients who underwent the hybrid approach (ACDF+ACCF). Radiographic measurements: C2–7 SVA

We had evaluated the radiological material on lateral radiographs and MRI before and after surgery. The cervical alignment defined as follows: positive = lordotic and negative = kyphotic. The T1 slope was defined as the angle between the superior endplate of T1 and a horizontal line on standing lateral radiograph. The C2–7 angle was defined as the angle of two lines parallel to the inferior endplates of C2 and C7 (Fig. 1). The local kyphotic angle (LKA) was measured between the superior endplate of upper end vertebrae and the inferior endplate of lower end vertebrae. The C2–C7 SVA was defined as the distance between the plumb line of C2 centroid and posterior superior corner of C7 (Fig. 2). The loss of intervertebral height more than 3 mm was defined as the cage subsidence between postoperative and final follow-up [13]. The C2–7 range of motion (ROM) was defined as the change in the maximal extension and flexion of cervical lateral radiographs. The worsening of postoperative local alignment was defined as the recurrence of kyphotic deformity after surgical correction. Moreover, the single increase of spinal cord on T2-weighted images and postoperative adjacent segment degeneration (ASD) was evaluated by MRI.

### Surgical procedure

The hybrid approach (ACDF+ACCF) was determined by patient’s physical condition, clinical signs and symptoms, and the compression location or levels of spinal cord on MRI or computed tomography (CT). Patients with ossification of the posterior vertebral body, kyphotic deformity, and multilevel spinal cord compression would be performed an anterior hybrid approach. The corpectomy was performed on the vertebra with posterior ossification, and the discectomy was performed on the segment of disc herniation. Blunt dissection was carried out in cervical muscles and reached responsible segments from inner side of the sternocleidomastoid. The anterior corpectomy bounded by the uncovertebral joint on both sides. We adequately removed the ossification of the posterior vertebral body for decompression of the spinal cord. An optimal polyether-ether-ketone (PEEK) cage and a titanium box cage with autogenous bone were implanted in an appropriate location with anterior fixation of plate system. The rupture bleeding of venous plexus had to be highly regarded. All patients required the utilization of a cervical collar for 3 or 4 weeks after surgery.

### Statistical analyses

Patient’s continuous variables and categorical variables were described as means and standard deviations, and frequencies and percentages, respectively. We used univariate analyses to assess the correlations between factors and postoperative outcome. Multivariate logistic regression analysis was performed to the variables with a *p* value less than 0.05 in univariate analysis. Mann-Whitney *U* test, independent *t* test, and chi-square test were performed for a comparison between improving of postoperative local alignment group and worsening of postoperative local alignment group. *p* value < 0.05 represents a statistically significant difference. All analyses were performed using version 22.0 of the SPSS software.

## Results

In Table 1, a series of variables about patient characteristics is shown. The good group had 66 patients with the recovery rates of JOA score greater than or equal to 50%, while the poor group had 20 patients with the recovery rates of JOA score less than 50%. Compared with the preoperative JOA score, there was a statistically significant improvement at the last follow-up (*p* < 0.05). However, a preoperative JOA score was not correlated to surgical outcome (*p* = 0.641). With the univariate analyses, the poor group had a significant difference in age (*p* < 0.001), duration of symptoms (*p* = 0.002), preoperative T1 slope angle (*p* = 0.001), change of local angle (CLA) (pre and last) (*p* < 0.001), LKA at last follow-up (*p* = 0.003), and worsening of postoperative local alignment (WPLA) (*p* = 0.032). Furthermore, Table 2 shows that age (OR = 1.09, 95% CI = 1.03–1.21, *p* = 0.017), duration of symptoms (OR = 4.64, 95% CI = 1.22–3.75, *p* = 0.001), T1 slope (OR = 1.02, 95% CI = 1.07–1.93, *p* < 0.001), and change of local kyphosis angle (OR = 10.67, 95% CI = 1.35–32.18, *p* = 0.003) were the risk factors of poor outcome after surgery.

**Table 1:** Comparison of patient characteristics between the good group and poor group

Variables	Good (*n* = 66)	Poor (*n* = 20)	*p* value
Age at surgery (years)	57.1 ± 8.8	63.4 ± 9.2	< 0.001
Female, *n* (%)	35 (53%)	11 (55%)	0.877
BMI (kg m^−2^)	23.5 ± 3.6	24.6 ± 4.1	0.309
Duration of symptoms (months)	11.6 ± 7.5	16.3 ± 11.8	0.002
T1 slope (°)	14.3 ± 2.4	17.8 ± 3.2	0.001
C2–7 SVA (mm)	17.6 ± 10.2	19.1 ± 9.4	0.266
Involved levels, *n* (%)			0.762
C2–5	13 (19.7%)	4 (20%)	
C3–6	31 (47%)	11 (55%)	
C4–7	22 (33.3%)	5 (25%)	
JOA score			
Preoperative	8.1 ± 2.2	7.5 ± 2.6	0.641
Postoperative	12.1 ± 3.7	10.9 ± 3.3	0.131
Last follow-up*	14.3 ± 2.9	10.1 ± 1.5	< 0.001
Recovery rate, %	64.7 ± 18.3	34.9 ± 10.5	< 0.001
C2–7 ROM (°)	38.2 ± 12.7	39.5 ± 13.0	0.891
C2–7 Cobb (°)			
Preoperative	− 10.7 ± 5.5	− 11.3 ± 6.2	0.219
Postoperative	7.1 ± 4.4	6.9 ± 5.0	0.774
Last follow-up	6.3 ± 5.7	5.6 ± 4.9	0.428
Change of C2–7 (pre and last)	12.4 ± 8.7	13.1 ± 9.5	0.138
LKA (°)			
Preoperative	− 13.7 ± 10.5	− 11.3 ± 9.2	0.736
Postoperative	5.1 ± 7.2	4.8 ± 6.3	0.552
Last follow-up	5.6 ± 3.9	0.3 ± 5.4	0.003
CLA (pre and last)	12.1 ± 7.7	8.7 ± 6.5	< 0.001
WPLA, *n* (%)	10 (15.1%)	7 (35%)	0.032
Implant subsidence > 3 mm, *n* (%)	6 (9%)	2 (10%)	0.817

*Abbreviations*: *BMI* body mass index, *T1* thoracic 1, *SVA* sagittal vertical axis, *JOA* Japanese Orthopaedic Association, *ROM* range of motion, *LKA* local kyphosis angle, *CLA* change of local angle, *WPLA* worsening of postoperative local alignment

*Significantly different from preoperative JOA score (*p* < 0.05)

**Table 2:** Multiple logistic regression analysis forecasted risk factors for the postoperative outcome

Variables	OR	95% CI	*p* value
Age at surgery (years)	1.09	1.03–1.21	0.017
T1 slope	1.02	1.07–1.93	< 0.001
Duration of symptoms (months)	4.64	1.22–3.75	0.001
LKA at last follow-up	1.73	0.88–2.69	0.118
CLA	10.67	1.35–32.18	0.003
WPLA	3.94	0.65–3.44	0.122

*Abbreviations*: *LKA* local kyphosis angle, *CLA* change of local angle, *WPLA* worsening of postoperative local alignment

In this study, the worsening of postoperative local alignment occurred in 17 patients (19.8%). Comparison of the improving group and worsening group showed that T1 slope (*p* < 0.001), preoperative C2–7 SVA (*p* < 0.001), adjacent segment degeneration (*p* = 0.01), and implant subsidence (*p* = 0.024) were significantly related to change of postoperative curvature (Table 3). Moreover, C5 palsy happened in 1 case which was functional recovery within 3 months after surgery, cerebrospinal fluid leakage happened in 5 cases, and temporary dysphagia happened in 7 cases in this study. None of them had a sequela.

**Table 3:** Comparison of postoperative local alignment between postoperative and last follow-up

Variables	Improving (*n* = 69)	Worsening (*n* = 17)	*p* value
Age (years)	62.5 ± 9.0	60.9 ± 10.2	0.332^a^
T1 slope (°)	18.7 ± 5.3	11.3 ± 4.2	< 0.001^b^
Preoperative C2–7 SVA (mm)	10.6 ± 8.8	24.9 ± 12.6	< 0.001^a^
ASD, *n* (%)	7 (10.1%)	6 (35.3%)	0.01^c^
Implant subsidence > 3 mm, *n* (%)	4 (5.8%)	4 (23.5%)	0.024^c^
Involved levels, *n* (%)			0.921^c^
C2–5	14	3	
C3–6	34	8	
C4–7	21	6	

*Abbreviations*: *ASD* adjacent segment degeneration

^a^Mann-Whitney *U* test

^b^Independent *t* test

^c^Chi-square test

## Discussion

Historically, early clinical intervention and optimal surgical approach have been recognized as the prerequisites of a good surgical outcome. This study found that the initial recovery is usually sensory which included limb numbness and zonesthesia of the chest. The motor function of limbs would gradually recover within 3 months after surgery, but it was generally difficult to recover completely. The recovery time of bladder function was long, and the recovery effect was poor. All kinds of risk factors affected the surgical outcome in patients with CSM and CK. Several articles have been published stating that predictors of postoperative outcome in patients with CSM included age at operation, duration of symptoms, and signal intensity of spinal cord [14, 15]. However, few studies paid close attention to clinical outcome after anterior hybrid surgery for patient with multilevel CSM and kyphosis deformity. Did cervical kyphosis impede the recovery of neurological function after surgery? Chavanne et al. [16] found that change of cervical alignment would lead to the increasing of intramedullary pressure, especially when cervical kyphotic deformity exceeded 21°. However, the correlation between correction of kyphosis alignment and postoperative outcome remained controversial [10, 17, 18].

An appropriate surgical approach was one of the important factors to achieve good postoperative outcome. However, the selection of surgical approach was the once controversial notion. Anterior approach could directly remove the lesion of compression of the spinal cord and correct more kyphotic angle. Unfortunately, anterior approaches were more vulnerable to complications which were C5 palsy, cerebrospinal fluid leakage, and temporary dysphagia [19]. Posterior approach relieved spinal cord compression according to the drift backward of the spinal cord and the enlarging of vertebral canal volume. Posterior approach would be an optimum selection when patients were accompanied by a consecutive ossification of posterior longitudinal ligament [20], whereas cervical kyphotic alignment reduced the space of drift backward of the spinal cord, which would influence clinical outcome. In previous study, Cabraja et al. [11] showed that anterior approach had a better restoration of cervical kyphosis than the posterior approach, and Suda et al. [21] showed that posterior laminoplasty was the best indication for patients with local kyphosis less than 13°. Furthermore, Yang et al. [22] showed a new style: anterior controllable ante-displacement fusion (ACAF), which could enlarge the volume of the spinal canal and simultaneously correct cervical kyphosis for patients with m-CSM and stenosis. In this study, an anterior hybrid approach was selected to remove ossification of the posterior vertebral body and correct kyphotic deformity. This method could not only directly decompress the spinal cord, but also avoid the occurrence of low fusion rate and implant translocation after multi-ACCF. Therefore, the selection of surgical approaches should be planned on an individual basis.

In this study, the change of local kyphotic angle between preoperative and last follow-up was associated with surgical outcome according to the JOA score, particularly corrective angle more than 10.2°. However, the change of C2–7 Cobb angle was not statistically significantly different between the good group and poor group. We considered that the cause was the occurrence of cervical “S” or reverse “S” type that lordosis and kyphosis existing side-by-side on the lateral films of cervical vertebra. Hence, the measurement of local kyphosis angle had greater meaning to the prevention of poor postoperative outcome. In previous study, Uchida et al. [10] explained also that correction of local cervical kyphosis was beneficial to improve the recovery of neurological function, whereas excessive correction of cervical kyphosis would give rise to short-term axial pain after surgery [23].

Not all patients could maintain the effect of surgical correction with the passage of time. In this study, there were seventeen patients with the worsening of postoperative local alignment as a result of each kind of suggestion factor. Although our research did not show that the worsening of postoperative local alignment was associated with poor outcome after surgery, the causes of worsening were necessary to study further. Lee et al. [24] found that one of the key factors affecting cervical spine sagittal balance was T1 slope. Patients with cervical kyphotic deformity required low T1 slope angle to compensate for maintaining the sagittal balance. In addition, Katsuura et al. [25] suggest that adjacent segment degeneration after anterior cervical fusion was also a key factor determining postoperative kyphotic change in the cervical fused segment. Our finding confirmed that the T1 slope and adjacent segment degeneration were associated with change of postoperative local alignment. The results expounded that surgical correction of cervical local kyphosis could not effectively improve the T1 slope angle, so recurrence of kyphotic deformity aimed to compensate for the balance of cervical alignment. Furthermore, we found also that implant subsidence and large preoperative C2–7 SVA were the important factors of worsening of postoperative local alignment. The implant subsidence would lead to the height difference of anterior and posterior margin in surgical segments. Frequent head-down tilt after surgery was the crucial reason of the subsidence in front of implant. Understanding and preventing risk factors could obtain maximum improvement in quality of life after surgery.

In our study, several limitations were worth mentioning. Firstly, this was a retrospective study with a relatively small number of patients and lacked long-term follow-up data for some patients. Secondly, the evaluation of surgical outcome only focused on improvement of JOA score and recovery rate, and neglected the evaluation according to the SF-36 scale and the HR-QOL scale. Thirdly, the relationship between the change of cervical kyphotic alignment and thoracolumbar or spino-pelvic parameters was not considered. Finally, the adjacent segment degeneration was evaluated only at the last follow-up, which may continue to occur in the future.

## Conclusion

Age at operation, duration of symptoms, T1 slope angle, and change of local kyphosis were the risk factors of clinical outcome in patients with CSM and cervical kyphosis. Furthermore, postoperative ASD, T1 slope, implant subsidence, and C2–7 Cobb were associated with recurrence of postoperative cervical kyphosis.

## Data Availability

Not applicable

## Abbreviations

JOA: Japanese Orthopaedic Association
SVA: Sagittal vertical axis
ASD: Adjacent segment degeneration
CSM: Cervical spondylotic myelopathy
CK: Cervical kyphosis
ACDF: Anterior cervical discectomy and fusion
ACCF: Anterior cervical corpectomy and fusion
LKA: Local kyphotic angle
CLA: Change of local angle
WPLA: Worsening of postoperative local alignment
ROM: Range of motion
AUC: Under the curve

## References

[1] Patwardhan AG, Khayatzadeh S, Havey RM, Voronov LI, Smith ZA, Kalmanson O, Ghanayem AJ, Sears W (2018). Cervical sagittal balance: a biomechanical perspective can help clinical practice. Eur Spine J.

[2] Ferch RD, Shad A, Cadoux-Hudson TAD, Teddy PJ (2004). Anterior correction of cervical kyphotic deformity: effects on myelopathy, neck pain, and sagittal alignment. J Neurosurg.

[3] Ao S, Liu Y, Wang Y, Zhang H, Leng H (2019). Cervical kyphosis in asymptomatic populations: incidence, risk factors, and its relationship with health-related quality of life. J Orthop Surg Res.

[4] Smith JS, Lafage V, Ryan DJ, Shaffrey CI, Schwab FJ, Patel AA, Brodke DS, Arnold PM, Riew KD, Traynelis VC (2013). Association of myelopathy scores with cervical sagittal balance and normalized spinal cord volume analysis of 56 preoperative cases from the AOSpine North America Myelopathy study. Spine.

[5] Scheer JK, Tang JA, Smith JS, Acosta FL, Protopsaltis TS, Blondel B, Bess S, Shaffrey CI, Deviren V, Lafage V (2013). Cervical spine alignment, sagittal deformity, and clinical implications: a review. Journal of Neurosurgery-Spine.

[6] Harrison DE, Cailliet R, Harrison DD, Janik TJ, Holland B (2002). A new 3-point bending traction method for restoring cervical lordosis and cervical manipulation: a nonrandomized clinical controlled trial. Arch Phys Med Rehabil.

[7] Emery SE, Bohlman HH, Bolesta MJ, Jones PK (1998). Anterior cervical decompression and arthrodesis for the treatment of cervical spondylotic myelopathy - two to seventeen-year follow-up. Journal of Bone and Joint Surgery-American Volume.

[8] Han K, Lu C, Li J, Xiong GZ, Wang B, Lv GH, Deng YW (2011). Surgical treatment of cervical kyphosis. Eur Spine J.

[9] Zhu B, Xu YL, Liu XG, Liu ZJ, Dang GT (2013). Anterior approach versus posterior approach for the treatment of multilevel cervical spondylotic myelopathy: a systemic review and meta-analysis. Eur Spine J.

[10] Uchida K, Nakajima H, Sato R, Yayama T, Mwaka ES, Kobayashi S, Baba H (2009). Cervical spondylotic myelopathy associated with kyphosis or sagittal sigmoid alignment: outcome after anterior or posterior decompression. Journal of Neurosurgery-Spine.

[11] Cabraja M, Abbushi A, Koeppen D, Kroppenstedt S, Woiciechowsky C (2010). Comparison between anterior and posterior decompression with instrumentation for cervical spondylotic myelopathy: sagittal alignment and clinical outcome. Neurosurg Focus.

[12] Furlan JC, Kalsi-Ryan S, Kailaya-Vasan A, Massicotte EM, Fehlings MG (2011). Functional and clinical outcomes following surgical treatment in patients with cervical spondylotic myelopathy: a prospective study of 81 cases clinical article. Journal of Neurosurgery-Spine.

[13] Van Jonbergen HPSM, Anderson PG (2005). Anterior cervical interbody fusion with a titanium box cage: early radiological assessment of fusion and subsidence. Spine J.

[14] Uchida K, Nakajima H, Sato R, Kokubo Y, Yayama T, Kobayashi S, Baba H (2005). Multivariate analysis of the neurological outcome of surgery for cervical compressive myelopathy. J Orthop Sci.

[15] Morio Y, Teshima R, Nagashima H, Nawata K, Yamasaki D, Nanjo Y (2001). Correlation between operative outcomes of cervical compression myelopathy and MRI of the spinal cord. Spine.

[16] Chavanne A, Pettigrew DB, Holtz JR, Dollin N, Kuntz C (2011). Spinal cord intramedullary pressure in cervical kyphotic deformity a cadaveric study. Spine.

[17] Kaptain GJ, Simmons NE, Replogle RE, Pobereskin L (2000). Incidence and outcome of kyphotic deformity following laminectomy for cervical spondylotic myelopathy. J Neurosurg.

[18] Rajshekhar V, Arunkumar MJ, Kumar SS (2003). Changes in cervical spine curvature after uninstrumented one- and two-level corpectomy in patients with spondylotic myelopathy. Neurosurgery.

[19] Hee HT, Majd ME, Holt RT, Whitecloud TS, Pienkowski D (2003). Complications of multilevel cervical corpectomies and reconstruction with titanium cages and anterior plating. J Spinal Disord Tech.

[20] Iwasaki M, Kawaguchi Y, Kimura T, Yonenobu K (2002). Long-term results of expansive laminoplasty for ossification of the posterior longitudinal ligament of the cervical spine: more than 10 years follow up. J Neurosurg.

[21] Suda K, Abumi K, Ito M, Shono Y, Kaneda K, Fujiya M (2003). Local kyphosis reduces surgical outcomes of expansive open-door laminoplasty for cervical spondylotic myelopathy. Spine.

[22] Yang HS, Yang Y, Shi JG, Guo YF, Sun JC, Shi GD, Zheng B (2018). Anterior controllable antedisplacement fusion as a choice for degenerative cervical kyphosis with stenosis: preliminary clinical and radiologic results. World Neurosurgery.

[23] Liu S, Yang DL, Zhao RY, Yang SD, Ma L, Wang H, Ding WY. Prevalence and risk factors of axial neck pain in patients undergoing multilevel anterior cervical decompression with fusion surgery. J Orthop Surg Res. 2019;14. 10.1186/s13018-019-1132-y PMC6450001 30947714

[24] Lee SH, Son ES, Seo EM, Suk KS, Kim KT (2015). Factors determining cervical spine sagittal balance in asymptomatic adults: correlation with spinopelvic balance and thoracic inlet alignment. Spine J.

[25] Katsuura A, Hukuda S, Saruhashi Y, Mori K (2001). Kyphotic malalignment after anterior cervical fusion is one of the factors promoting the degenerative process in adjacent intervertebral levels. Eur Spine J.

